# A very short O—H⋯O hydrogen bond in the structure of clozapinium hydrogen bis­(3,5-di­nitro­benzoate)

**DOI:** 10.1107/S2056989020012268

**Published:** 2020-09-11

**Authors:** Mohammed A. E. Shaibah, Channappa N. Kavitha, Hemmige S. Yathirajan, Sabine Foro, Christopher Glidewell

**Affiliations:** a Department of Studies in Chemistry, University of Mysore, Manasagangotri, Mysuru 570 006, India; b Department of Chemistry, Maharani’s Science College for Women, Mysuru 570 001, India; c Institute of Materials Science, Darmstadt University of Technology, Alarich-Weiss-Strasse 2, D-64287 Darmstadt, Germany; d School of Chemistry, University of St Andrews, St Andrews, Fife KY16 9ST, UK

**Keywords:** clozapine, crystal structure, mol­ecular conformation, hydrogen bonding, very short hydrogen bonds, supra­molecular assembly

## Abstract

In the title salt {systematic name: 4-[6-chloro-2,9-di­aza­tri­cyclo­[9.4.0.0^3,8^]penta­deca-1(15),3(8),4,6,9,11,13-heptaen-10-yl]-1-methyl­piperazin-1-ium 3,5-di­nitro­benzoate–3,5-di­nitro­benzic acid (1/1)}, C_18_H_20_ClN_4_^+^·C_7_H_3_N_2_O_6_^−^·C_7_H_4_N_2_O_6_, there is a very short, asymmetric, O—H⋯O hydrogen bond [O⋯O = 2.453 (3) Å] within the anion. The oxygen atoms of one of the nitro groups of the anion are disordered over two sets of sites having occupancies of 0.56 (3) and 0.44 (3). The fused tricyclic portion of the cation adopts a butterfly conformation, with a dihedral angle of 45.59 (6)° between the planes of the two aryl rings. In the crystal, a combination of O—H⋯O, N—H⋯O and C—H⋯O hydrogen bonds links the component species into a three-dimensional framework. Comparisons are made with the structures of some related compounds.

## Abstract 2

The title compound features a very short, but asymmetric, O—H⋯O hydrogen bond having an O⋯O distance of 2.452 (3) Å within the anion.

## Chemical context   

Clozapine, 8-chloro-11-(4-methyl­piperazin-1-yl)-5*H*-dibenzo[*b*,*e*][1,4]diazepine, C_18_H_19_ClN_4_, is a well established medication for the treatment of schizophrenia, often preferred over other treatments because of the generally lower incidence of adverse side effects (Breier *et al.*, 1994 ▸). The structure of the free base has been reported (Petcher & Weber, 1976 ▸; Fillers & Hawkinson, 1982 ▸), along with those of a few salts (Fillers & Hawkinson, 1982 ▸; Kaur *et al.*, 2015 ▸). Among the latter is the 1:1 salt formed by the reaction of clozapine with an equimolar qu­antity of 3,5-di­nitro­benzoic acid in methanol followed by slow crystallization from di­methyl­sulfoxide solution, when a DMSO monosolvate of the 1:1 salt was obtained (Kaur *et al.*, 2015 ▸).
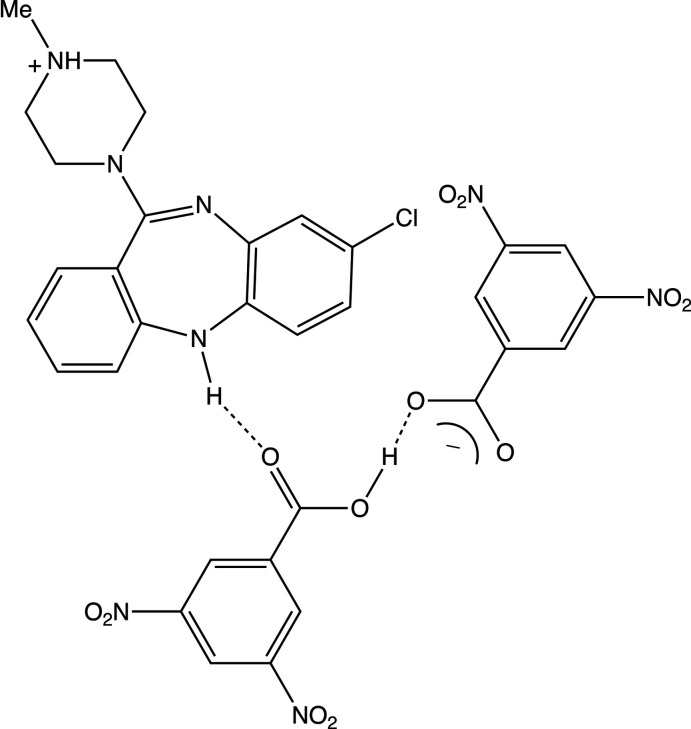


We have now found that repetition of this process but with the substitution of di­methyl­sulfoxide by a 1:1 mixture of chloro­form and methanol gives the solvent-free 1:2 acid salt chlozapinium hydrogen bis(3,5-di­nitro­benzoate), (I), whose structure we report here along with comparisons between the structure of (I) and those of both the solvated 1:1 salt, (II), (Kaur *et al.*, 2015 ▸) and the 1:2 acid salt, (III), formed between 3,5-di­nitro­benzoic acid and the anti­psychotic agent chlorprothixene, 3-(2-chloro-9*H*-thioxanthen-9-yl)-*N*,*N*-di­methyl­propan-1-amine (Shaibah *et al.*, 2019 ▸).

## Structural commentary   

**Figure 1:**
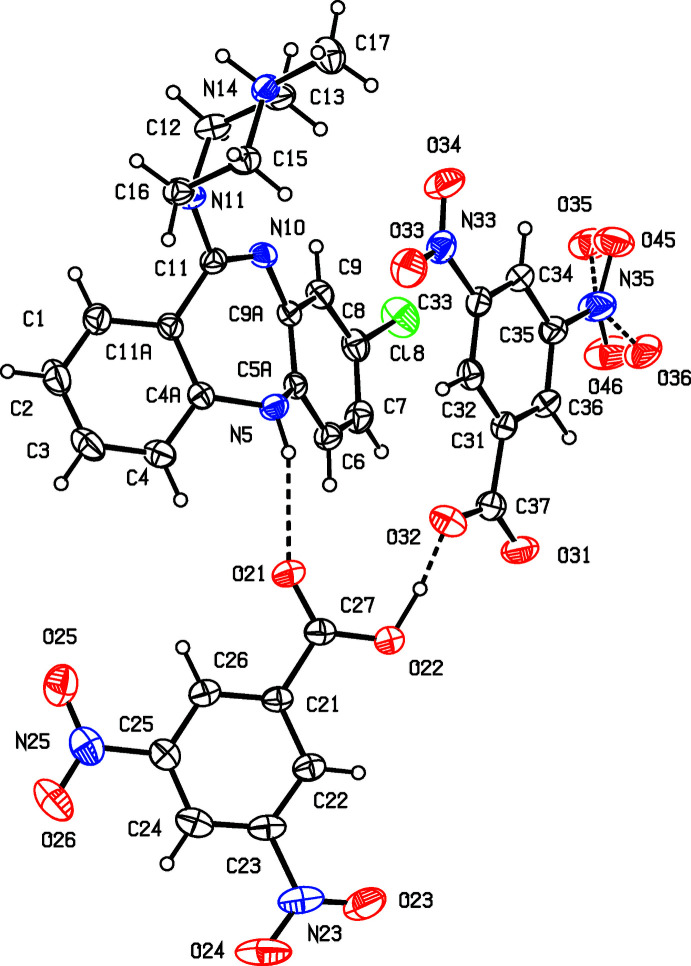
The mol­ecular structure of (I), showing displacement ellipsoids drawn at the 30% probability level and hydrogen bonds (dashed lines) within the asymmetric unit.

Compound (I) is an acid salt, *i.e*., the asymmetric unit contains one C_18_H_20_ClN_4_^+^ clozapinium cation and one C_14_H_7_N_4_O_12_^−^ hydrogen bis­(3,5-dinotrobenzoate) anion (Fig. 1 ▸). An alternative description is one cation, one 3,5-di­nitro­benzote anion and one neutral mol­ecule of 3,5-di­nitro­benzoic acid, *i.e*., C_18_H_20_ClN_4_^+^·(C_7_H_3_N_2_O_6_)^−^·(C_7_H_4_N_2_O_6_). The –CO_2_H and –CO_2_^−^ groups in the anion are linked by a very short O22—H22*A*⋯O32 hydrogen bond (Table 1 ▸) (Speakman, 1972 ▸; Emsley, 1980 ▸; Gerlt *et al.*, 1997 ▸) but, although it is nearly linear [169 (3)°], it is not symmetric as the two independent O—H distances are significantly different [O22—H22*A* = 1.11 (4); H22*A*⋯O32 = 1.35 (4) Å]. There is a similarly short O—H⋯O hydrogen bond in the corresponding species of the chlorprothixene salt (III) (Shaibah *et al.*, 2019 ▸), where the O⋯O distance, 2.4197 (15) Å, is slightly shorter than that found here for (I), while the difference between the two independent O—H distances is about 50% higher in (III) as compared to (I). For (I) it is possible to select a compact asymmetric unit in which the components are linked by O—H⋯O and N—H⋯O hydrogen bonds (Table 1 ▸, Fig. 1 ▸). Within this asymmetric unit, there are also two fairly short C—H⋯O contacts. That involving atom C4 has a small C—H⋯O angle, and so it probably not structurally significant (Wood *et al.*, 2009 ▸), while for that involving atom C13, the H⋯O distance is not significantly shorter that the sum of the van der Waals radii (Rowland & Taylor, 1996 ▸). These are both probably better regarded as adventitious contacts rather than as structurally significant hydrogen bonds.

One of the nitro groups of the anion, that attached to C35, is disordered over two sets of atomic sites, with occupancies of 0.56 (3) and 0.44 (3) for the oxygen atoms. The major and minor disorder components make dihedral angles with the adjacent aryl ring of 17.2 (8) and 19.4 (8)°, with a dihedral angle between their own planes of 36.5 (14)°, so that these components are rotated out of the plane of the aryl ring in opposite senses.

In the C_18_H_20_ClN_4_^+^ cation of (I), the fused tricyclic component adopts a butterfly conformation with a dihedral angle of 45.59 (6)° between the planes of the two outer aryl rings. The piperazine ring adopts a chair conformation, as indicated by the value of the ring-puckering angle θ = 176.0 (3)°, as calculated for the atom sequence N11/C12/C13/N14/C15/C16: for an idealized chair form this angle takes a value of either zero or 180° (Boeyens, 1978 ▸). The site of protonation is the methyl­ated atom N14 where the methyl substituent occupies the equatorial site (Fig. 1 ▸). The geometry at the other N atom in this ring, atom N11, is nearly planar: the sum of the C—N—C angles at N11 is 351.9°, as compared with 344.1° at N14, while the displacements of these N atoms from the planes of the adjacent three C atoms are 0.449 (3) Å for N14 and 0.236 (2) Å for N11.

## Supra­molecular features   

**Figure 2:**
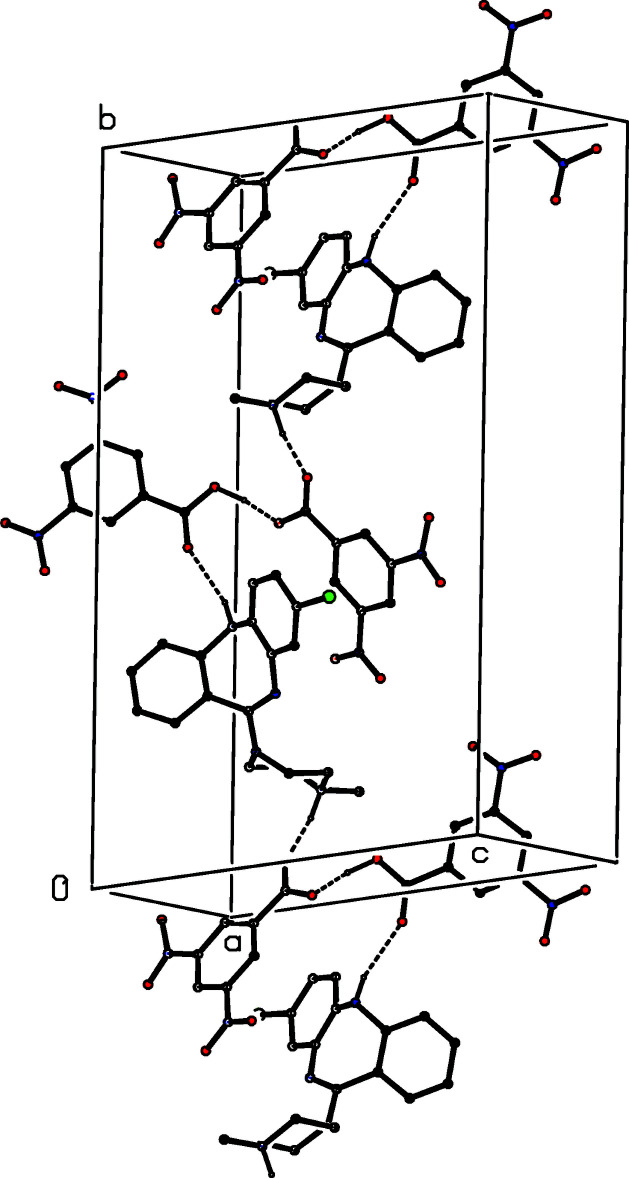
Part of the crystal structure of (I) showing the formation of a hydrogen-bonded 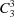
(17) chain running parallel to [010]. Hydrogen bonds are drawn as dashed lines. For the sake of clarity, the H atoms bonded to C atoms have all been omitted.

**Figure 3:**
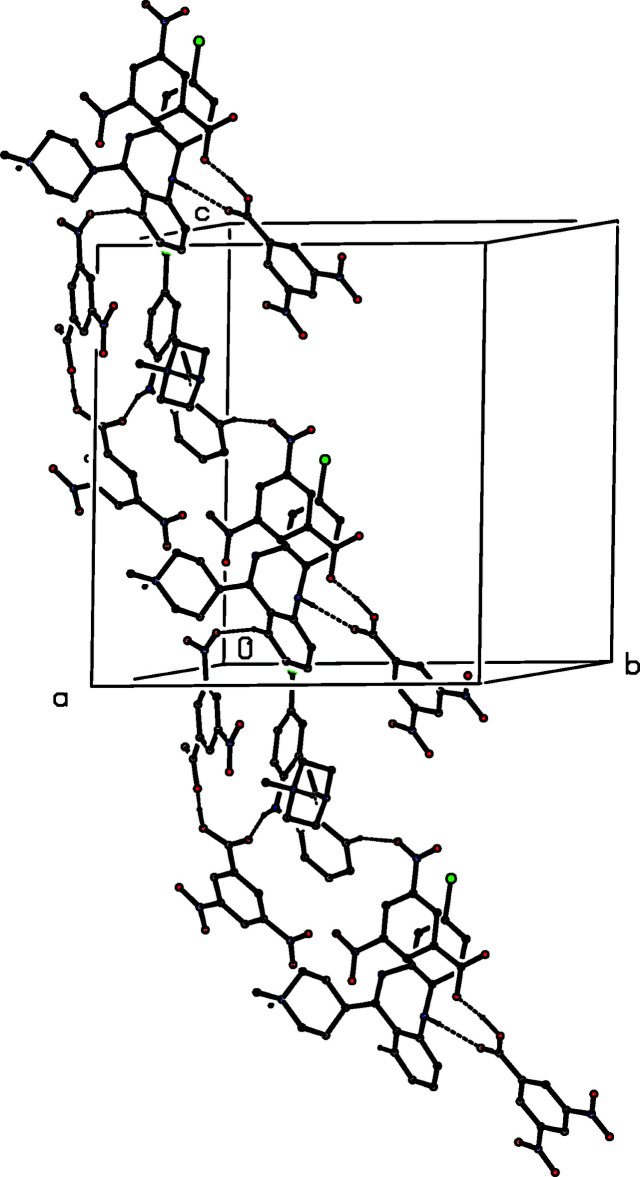
Part of the crystal structure of (I) showing the formation of a hydrogen-bonded 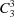
(17) chain running parallel to [101]. Hydrogen bonds are drawn as dashed lines. For the sake of clarity, the H atoms bonded to those C atoms that are not involved in the motif shown have been omitted.

**Figure 4:**
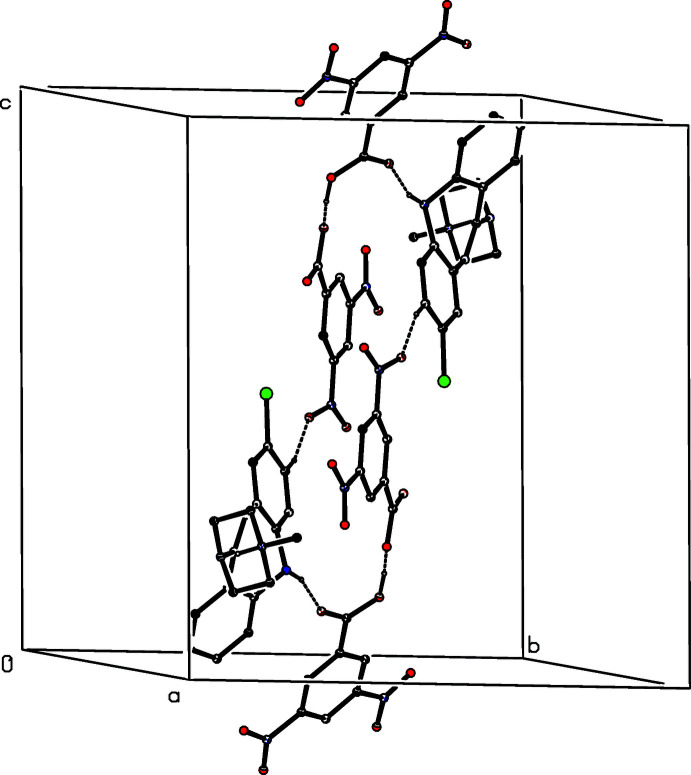
Part of the crystal structure of (I) showing the formation of a hydrogen-bonded 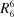
(34) ring. Hydrogen bonds are drawn as dashed lines. For the sake of clarity, the H atoms bonded to those C atoms that are not involved in the motif shown have been omitted.

Aggregates of the type defining the selected asymmetric unit (Fig. 1 ▸) are linked by a combination of one N—H⋯O, one O—H⋯O and two C—H⋯O hydrogen bonds (Table 1 ▸) to form a three-dimensional network: since both disorder components participate in similar hydrogen bonds, it is necessary to consider only the inter­actions involving the major component. The formation of the hydrogen-bonded network is readily analysed in terms of three simple sub-structures (Ferguson *et al.*, 1998*a* ▸,*b* ▸; Gregson *et al.*, 2000 ▸), in which the asymmetric unit aggregates are linked in different ways, each utilizing just one of the three inter-aggregate hydrogen bonds. The N14–H14⋯O31^i^ (see Table 1 ▸ for symmetry codes) hydrogen bond links the aggregates into a 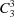
(17) (Etter, 1990 ▸; Etter *et al.*, 1990 ▸; Bernstein *et al.*, 1995 ▸) chain running parallel to the [010] direction (Fig. 2 ▸). In the second sub-structure, the C1—H1⋯O35^ii^ hydrogen bond links the aggregates into another 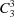
(17) chain, this time running parallel to the [101] direction (Fig. 3 ▸). In the final sub-structure, the C7—H7⋯O36^iii^ hydrogen bond links inversion-related pairs of aggregates into a cyclic centrosymmetric system characterized by an 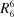
(34) motif (Fig. 4 ▸). The combination of the chains along [010] and [101] generates a complex sheet lying parallel to (10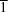), and adjacent sheets are linked by the 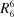
(34) motif, thereby generating a three-dimensional array.

## Database survey   

Here we briefly compare the salient features of the structure of compound (I), with those of some related structures. As noted above (Section 2), the O⋯O distances in the anion of the chloro­thixene salt (III) (Shaibah *et al.*, 2019 ▸), is slightly shorter than that found here for compound (I). Although the O⋯O distances in (I) and (III) are very short, some even shorter distances have been reported, some below 2.40 Å. One of the simplest organic compounds to display such a short distance is the enol form, Me_3_C(OH)=C(CN)COCMe_3_, of the 1,3 diketone 4-cyano 2,2,6,6-tetra­methyl3,5-hepta­nedione, where the intra­molecular O—H⋯O hydrogen bond has an O⋯O distance of 2.3936 (15) Å (Belot *et al.*, 2004 ▸), while the corresponding distances in some cyclic phosphate derivatives are reported to be as low as 2.368 (4) Å (Kumara Swamy *et al.*, 2001 ▸).

The dihedral angles between the planes of the pendent aryl rings in the fused tricyclic portion of various clozapine derivatives show some curious variations. In the free base (Fillers & Hawkinson, 1982 ▸) this angle is 67.3° [unfortunately, the atomic coordinates retrieved from the CSD (Groom *et al.*, 2016 ▸) have no s.u. values] and in the monohydrate (CSD refcode DEHBUP; the publication cited in the CSD could not be traced) and the methanol solvate (Verma *et al.*, 2018 ▸), the corresponding angles are 63.4 and 56.1°, respectively. In the 1:1 salt formed with 3,5-di­nitro­benzoic acid (II), this angle is 62.21 (11)° (Kaur *et al.*, 2015 ▸), fairly similar to the values of 60.97 (9) and 59.07 (16)° in the 1:1 salts formed with maleic and 2-hy­droxy­benzoic acids, respectively (Kaur *et al.*, 2015 ▸). In the di(hydro­bomide) salt, the angle is 52.3° (Fillers & Hawkinson, 1982 ▸), while in the ethanol solvate of clozapine *N*-oxide, the corresponding angle is 56.2° (van der Peet *et al.*, 2018 ▸). There are, at present, too few data for any pattern to be discernible in the variation of this dihedral angle.

The hydrogen-bonded supra­molecular assembly of compound (I) is three dimensional (Section 3, above), but in the solvated 1:1 salt (II), the hydrogen-bonded ion pairs are linked into chains by a π–π stacking inter­action (Kaur *et al.*, 2015 ▸). There are no hydrogen bonds in the structure of clozapine itself (Fillers & Hawkinson, 1982 ▸), but in the monohydrate (DEHBUP), a combination of one N—H⋯O hydrogen bond and two O—H⋯N hydrogen bonds links the components into a chain of rings. In the methanol solvate of clozapine (Verma *et al.*, 2018 ▸), the components are linked by an O—H⋯N hydrogen bond, but with no further aggregation. In the hydrogenmaleate and 2-hy­droxy­benzoate salts, multiple hydrogen bonds generate sheets and a three-dimensional supra­molecular network, respectively (Kaur *et al.*, 2015 ▸), while in the di(hydro­bromide) salt, the ions are linked into chains by N—H⋯Br hydrogen bonds (Fillers & Hawkinson, 1982 ▸).

## Synthesis and crystallization   

Clozapine (100 mg, 0.31 mmol) and 3,5-di­nitro­benzoic acid (66 mg, 0.31mmol) were dissolved in methanol (10 ml), and this mixture was then stirred at 333 K for a few minutes. The solution was permitted to cool to room temperature and the resulting crystals were then collected by filtration and dried over P_2_O_5_. Crystals of (I) suitable for single-crystal X-ray diffraction were obtained by slow evaporation, at room temperature and in the presence of air, of a solution in the mixed solvents of chloro­form and methanol (initial composition 1:1, *v*/*v*); m.p. 494–497 K.

## Refinement   

Crystal data, data collection and refinement details are summarized in Table 2 ▸. All H atoms were located in difference maps. The H atoms bonded to C atoms were then treated as riding atoms in geometrically idealized positions with C—H distances of 0.93 Å (aromatic), 0.96 Å (CH_3_) or 0.97 Å (CH_2_), and with *U*_iso_(H) = 1.2*U*_eq_(C) or 1.5*U*_eq_(methyl C); the CH_3_ group was permitted to rotate but not to tilt. For the H atoms bonded to N or O atoms, the atomic coordinates were refined with *U*_iso_(H) = 1.2*U*_eq_(N) or 1.5*U*_eq_(O). For the minor disorder component, the N—O distances and the 1,3-non-bonded O⋯O distances were restrained to be the same of the corresponding distances in the major component, subject to s.u. values of 0.01 and 0.02 Å, respectively. In addition, a similarity restraint was applied to the disordered O-atom sites and for each of the disorder components, the C—NO_2_ fragment was restrained to be planar. Subject to these conditions, the refined disorder occupancies are 0.56 (3) and 0.44 (3).

## Supplementary Material

Crystal structure: contains datablock(s) global, I. DOI: 10.1107/S2056989020012268/hb7944sup1.cif

Structure factors: contains datablock(s) I. DOI: 10.1107/S2056989020012268/hb7944Isup2.hkl

Supporting information file. DOI: 10.1107/S2056989020012268/hb7944Isup3.cml

CCDC reference: 2027224

Additional supporting information:  crystallographic information; 3D view; checkCIF report

## Figures and Tables

**Table 1:** Hydrogen-bond geometry (Å, °)

*D*—H⋯*A*	*D*—H	H⋯*A*	*D*⋯*A*	*D*—H⋯*A*
N5—H5⋯O21	0.88 (3)	2.23 (3)	3.079 (3)	162 (3)
O22—H22*A*⋯O32	1.11 (4)	1.35 (4)	2.453 (3)	169 (3)
C4—H4⋯O21	0.93	2.48	3.280 (4)	144
C13—H13*A*⋯O34	0.97	2.60	3.539 (4)	164
N14—H14⋯O31^i^	1.00 (3)	1.70 (3)	2.689 (3)	169 (3)
C1—H1⋯O35^ii^	0.93	2.39	3.292 (13)	164
C7—H7⋯O36^iii^	0.93	2.34	3.242 (13)	163
C7—H7⋯O46^iii^	0.93	2.37	3.253 (14)	159

Symmetry codes: (i) 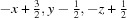; (ii) 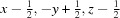; (iii) 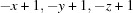.

**Table 2:** Experimental details

Crystal data	
Chemical formula	C_18_H_20_ClN_4_^+^·C_7_H_3_N_2_O_6_^−^·C_7_H_4_N_2_O_6_
*M*_r_	751.07
Crystal system, space group	Monoclinic, *P*2_1_/*n*
Temperature (K)	296
*a*, *b*, *c* (Å)	7.4102 (6), 24.629 (2), 18.446 (1)
β (°)	98.478 (6)
*V* (Å^3^)	3329.7 (4)
*Z*	4
Radiation type	Mo *K*α
μ (mm^−1^)	0.19
Crystal size (mm)	0.36 × 0.24 × 0.20

Data collection	
Diffractometer	Oxford Diffraction Xcalibur Sapphire CCD detector
Absorption correction	Multi-scan (*CrysAlis RED*; Oxford Diffraction, 2009 ▸)
*T*_min_, *T*_max_	0.914, 0.962
No. of measured, independent and observed [*I* > 2σ(*I*)] reflections	13700, 6870, 3811
*R*_int_	0.036
(sin θ/λ)_max_ (Å^−1^)	0.629

Refinement	
*R*[*F* ^2^ > 2σ(*F* ^2^)], *wR*(*F* ^2^), *S*	0.062, 0.133, 1.04
No. of reflections	6870
No. of parameters	507
No. of restraints	17
H-atom treatment	H atoms treated by a mixture of independent and constrained refinement
Δρ_max_, Δρ_min_ (e Å^−3^)	0.16, −0.20

Computer programs: *CrysAlis CCD* and *CrysAlis RED* (Oxford Diffraction, 2009 ▸), *SHELXT* (Sheldrick, 2015*a* ▸), *SHELXL2014* (Sheldrick, 2015*b* ▸) and *PLATON* (Spek, 2020 ▸).

## supplementary crystallographic information

### Crystal data

**Table:**

C_18_H_20_ClN_4_^+^·C_7_H_3_N_2_O_6_^−^·C_7_H_4_N_2_O_6_	*F*(000) = 1552
*M**_r_* = 751.07	*D*_x_ = 1.498 Mg m^−^^3^
Monoclinic, *P*2_1_/*n*	Mo *K*α radiation, λ = 0.71073 Å
*a* = 7.4102 (6) Å	Cell parameters from 7183 reflections
*b* = 24.629 (2) Å	θ = 2.8–27.9°
*c* = 18.446 (1) Å	µ = 0.19 mm^−^^1^
β = 98.478 (6)°	*T* = 296 K
*V* = 3329.7 (4) Å^3^	Needle, red
*Z* = 4	0.36 × 0.24 × 0.20 mm

### Data collection

**Table:**

Oxford Diffraction Xcalibur Sapphire CCD detector diffractometer	6870 independent reflections
Radiation source: Enhance (Mo) X-ray Source	3811 reflections with *I* > 2σ(*I*)
Graphite monochromator	*R*_int_ = 0.036
ω scans	θ_max_ = 26.6°, θ_min_ = 2.8°
Absorption correction: multi-scan (CrysAlis RED; Oxford Diffraction, 2009)	*h* = −9→8
*T*_min_ = 0.914, *T*_max_ = 0.962	*k* = −18→30
13700 measured reflections	*l* = −20→23

### Refinement

**Table:**

Refinement on *F*^2^	Primary atom site location: dual
Least-squares matrix: full	Secondary atom site location: difference Fourier map
*R*[*F*^2^ > 2σ(*F*^2^)] = 0.062	Hydrogen site location: mixed
*wR*(*F*^2^) = 0.133	H atoms treated by a mixture of independent and constrained refinement
*S* = 1.04	*w* = 1/[σ^2^(*F*_o_^2^) + (0.0452*P*)^2^ + 1.0626*P*] where *P* = (*F*_o_^2^ + 2*F*_c_^2^)/3
6870 reflections	(Δ/σ)_max_ < 0.001
507 parameters	Δρ_max_ = 0.16 e Å^−^^3^
17 restraints	Δρ_min_ = −0.19 e Å^−^^3^

### Special details

**Table:**

Geometry. All esds (except the esd in the dihedral angle between two l.s. planes) are estimated using the full covariance matrix. The cell esds are taken into account individually in the estimation of esds in distances, angles and torsion angles; correlations between esds in cell parameters are only used when they are defined by crystal symmetry. An approximate (isotropic) treatment of cell esds is used for estimating esds involving l.s. planes.

### Fractional atomic coordinates and isotropic or equivalent isotropic displacement parameters (Å^2^)

**Table:**

	*x*	*y*	*z*	*U*_iso_*/*U*_eq_	Occ. (<1)
C1	0.3444 (4)	0.23128 (13)	0.08513 (15)	0.0519 (8)	
H1	0.3445	0.1944	0.0957	0.062*	
C2	0.2571 (4)	0.24929 (16)	0.01823 (17)	0.0659 (9)	
H2	0.1993	0.2247	−0.0157	0.079*	
C3	0.2559 (4)	0.30335 (16)	0.00210 (16)	0.0650 (9)	
H3	0.2007	0.3155	−0.0436	0.078*	
C4	0.3359 (4)	0.33980 (13)	0.05311 (15)	0.0525 (8)	
H4	0.3326	0.3767	0.0421	0.063*	
C4A	0.4220 (3)	0.32228 (11)	0.12132 (13)	0.0383 (6)	
N5	0.5009 (3)	0.36063 (10)	0.17327 (12)	0.0437 (6)	
H5	0.492 (4)	0.3941 (12)	0.1564 (15)	0.052*	
C5A	0.4443 (3)	0.35994 (11)	0.24375 (13)	0.0374 (6)	
C6	0.3781 (4)	0.40729 (12)	0.27020 (15)	0.0458 (7)	
H6	0.3563	0.4371	0.2391	0.055*	
C7	0.3434 (4)	0.41167 (13)	0.34144 (16)	0.0519 (8)	
H7	0.3007	0.4440	0.3586	0.062*	
C8	0.3733 (4)	0.36715 (13)	0.38635 (15)	0.0487 (7)	
Cl8	0.34891 (13)	0.37265 (4)	0.47895 (4)	0.0768 (3)	
C9	0.4298 (3)	0.31860 (12)	0.36041 (14)	0.0463 (7)	
H9	0.4435	0.2884	0.3910	0.056*	
C9A	0.4666 (3)	0.31418 (11)	0.28869 (14)	0.0379 (6)	
N10	0.5458 (3)	0.26494 (9)	0.27155 (11)	0.0410 (5)	
C11	0.5353 (3)	0.24542 (11)	0.20636 (14)	0.0396 (6)	
C11A	0.4322 (3)	0.26698 (11)	0.13697 (13)	0.0391 (6)	
N11	0.6093 (3)	0.19388 (9)	0.20017 (11)	0.0451 (6)	
C12	0.6660 (4)	0.16212 (12)	0.26609 (15)	0.0524 (8)	
H12A	0.6413	0.1241	0.2553	0.063*	
H12B	0.5939	0.1729	0.3035	0.063*	
C13	0.8645 (4)	0.16879 (12)	0.29558 (15)	0.0524 (8)	
H13A	0.8889	0.2062	0.3105	0.063*	
H13B	0.8964	0.1457	0.3382	0.063*	
N14	0.9768 (3)	0.15369 (10)	0.23769 (13)	0.0472 (6)	
H14	0.947 (4)	0.1154 (12)	0.2223 (14)	0.057*	
C15	0.9216 (4)	0.18776 (12)	0.17106 (15)	0.0508 (7)	
H15A	0.9913	0.1769	0.1329	0.061*	
H15B	0.9492	0.2255	0.1828	0.061*	
C16	0.7201 (4)	0.18191 (12)	0.14324 (15)	0.0471 (7)	
H16A	0.6868	0.2064	0.1023	0.057*	
H16B	0.6954	0.1451	0.1257	0.057*	
C17	1.1766 (4)	0.15711 (16)	0.2639 (2)	0.0800 (11)	
H17A	1.2074	0.1934	0.2803	0.120*	
H17B	1.2425	0.1480	0.2245	0.120*	
H17C	1.2086	0.1322	0.3037	0.120*	
C21	0.2811 (4)	0.54057 (11)	0.01705 (14)	0.0419 (7)	
C22	0.2657 (4)	0.59602 (12)	0.00575 (16)	0.0492 (7)	
H22	0.3254	0.6201	0.0400	0.059*	
C23	0.1607 (4)	0.61492 (13)	−0.05705 (17)	0.0535 (8)	
C24	0.0736 (4)	0.58098 (14)	−0.11005 (17)	0.0572 (8)	
H24	0.0046	0.5945	−0.1524	0.069*	
C25	0.0928 (4)	0.52651 (13)	−0.09794 (15)	0.0484 (7)	
C26	0.1939 (4)	0.50581 (12)	−0.03532 (15)	0.0456 (7)	
H26	0.2033	0.4684	−0.0284	0.055*	
C27	0.3916 (4)	0.51740 (13)	0.08452 (16)	0.0473 (7)	
O21	0.3873 (3)	0.46873 (9)	0.09583 (11)	0.0678 (6)	
O22	0.4857 (3)	0.55163 (8)	0.12759 (12)	0.0570 (6)	
H22A	0.572 (5)	0.5330 (14)	0.1755 (19)	0.085*	
N23	0.1434 (5)	0.67388 (13)	−0.0680 (2)	0.0740 (9)	
O23	0.2158 (4)	0.70360 (11)	−0.0192 (2)	0.1043 (10)	
O24	0.0524 (5)	0.68988 (11)	−0.12377 (16)	0.1095 (10)	
N25	−0.0005 (4)	0.48816 (15)	−0.15269 (16)	0.0669 (8)	
O25	0.0063 (4)	0.44003 (12)	−0.13688 (13)	0.0836 (8)	
O26	−0.0774 (3)	0.50674 (12)	−0.21036 (14)	0.0935 (9)	
C31	0.7597 (4)	0.47059 (12)	0.34692 (15)	0.0446 (7)	
C32	0.8228 (4)	0.42264 (12)	0.32110 (15)	0.0463 (7)	
H32	0.8254	0.4179	0.2713	0.056*	
C33	0.8817 (4)	0.38203 (11)	0.37005 (15)	0.0447 (7)	
C34	0.8818 (4)	0.38730 (12)	0.44445 (16)	0.0496 (7)	
H34	0.9216	0.3594	0.4769	0.060*	
C35	0.8204 (4)	0.43570 (12)	0.46830 (15)	0.0503 (7)	
C36	0.7589 (4)	0.47737 (12)	0.42137 (15)	0.0505 (7)	
H36	0.7173	0.5096	0.4394	0.061*	
C37	0.6800 (4)	0.51417 (14)	0.29415 (17)	0.0507 (8)	
O31	0.6202 (3)	0.55554 (9)	0.31888 (12)	0.0689 (6)	
O32	0.6772 (3)	0.50299 (9)	0.22654 (12)	0.0653 (6)	
N33	0.9440 (3)	0.33021 (11)	0.34261 (16)	0.0565 (7)	
O33	0.9549 (3)	0.32681 (10)	0.27753 (13)	0.0774 (7)	
O34	0.9802 (4)	0.29336 (9)	0.38628 (13)	0.0768 (7)	
N35	0.8168 (5)	0.44262 (13)	0.54739 (15)	0.0740 (9)	0.44 (3)
O35	0.830 (4)	0.4023 (5)	0.5865 (10)	0.086 (5)	0.44 (3)
O36	0.816 (3)	0.4907 (3)	0.5686 (6)	0.078 (3)	0.44 (3)
N45	0.8168 (5)	0.44262 (13)	0.54739 (15)	0.0740 (9)	0.56 (3)
O45	0.900 (2)	0.4100 (6)	0.5902 (8)	0.084 (4)	0.56 (3)
O46	0.712 (3)	0.4787 (6)	0.5660 (5)	0.098 (4)	0.56 (3)

### Atomic displacement parameters (Å^2^)

**Table:**

	*U*^11^	*U*^22^	*U*^33^	*U*^12^	*U*^13^	*U*^23^
C1	0.0503 (18)	0.0489 (19)	0.0549 (18)	0.0007 (16)	0.0026 (14)	−0.0084 (15)
C2	0.059 (2)	0.076 (3)	0.057 (2)	0.007 (2)	−0.0099 (16)	−0.0188 (19)
C3	0.064 (2)	0.084 (3)	0.0425 (17)	0.017 (2)	−0.0079 (15)	−0.0011 (18)
C4	0.0569 (19)	0.054 (2)	0.0453 (17)	0.0110 (17)	0.0035 (14)	0.0052 (15)
C4A	0.0344 (15)	0.0427 (17)	0.0383 (14)	0.0025 (13)	0.0067 (12)	−0.0013 (13)
N5	0.0507 (14)	0.0357 (14)	0.0448 (13)	−0.0013 (12)	0.0074 (11)	0.0039 (11)
C5A	0.0299 (14)	0.0396 (16)	0.0412 (15)	−0.0018 (13)	0.0000 (11)	−0.0033 (13)
C6	0.0411 (16)	0.0384 (17)	0.0571 (18)	0.0022 (14)	0.0049 (13)	−0.0026 (14)
C7	0.0440 (17)	0.0478 (19)	0.064 (2)	0.0034 (16)	0.0089 (15)	−0.0175 (16)
C8	0.0429 (17)	0.060 (2)	0.0429 (16)	0.0014 (16)	0.0059 (13)	−0.0100 (15)
Cl8	0.0832 (6)	0.0991 (7)	0.0497 (5)	0.0122 (6)	0.0154 (4)	−0.0144 (5)
C9	0.0409 (17)	0.0518 (19)	0.0443 (16)	0.0026 (15)	0.0006 (13)	−0.0010 (14)
C9A	0.0333 (14)	0.0371 (16)	0.0411 (15)	0.0009 (13)	−0.0021 (11)	−0.0010 (12)
N10	0.0389 (13)	0.0390 (14)	0.0441 (13)	0.0087 (11)	0.0027 (10)	−0.0006 (11)
C11	0.0351 (15)	0.0352 (16)	0.0489 (16)	0.0000 (13)	0.0069 (12)	0.0018 (13)
C11A	0.0324 (14)	0.0413 (17)	0.0430 (15)	0.0024 (13)	0.0031 (12)	−0.0024 (13)
N11	0.0502 (14)	0.0387 (14)	0.0478 (13)	0.0101 (12)	0.0113 (11)	0.0044 (11)
C12	0.063 (2)	0.0390 (17)	0.0585 (18)	0.0109 (16)	0.0202 (15)	0.0089 (14)
C13	0.070 (2)	0.0381 (17)	0.0485 (17)	0.0090 (16)	0.0081 (15)	−0.0001 (14)
N14	0.0435 (14)	0.0375 (14)	0.0601 (15)	−0.0027 (12)	0.0058 (12)	0.0006 (12)
C15	0.0501 (18)	0.0459 (18)	0.0577 (18)	0.0016 (15)	0.0125 (14)	0.0069 (15)
C16	0.0513 (18)	0.0410 (17)	0.0497 (16)	0.0081 (15)	0.0095 (14)	−0.0012 (13)
C17	0.047 (2)	0.085 (3)	0.102 (3)	−0.011 (2)	−0.0084 (18)	0.011 (2)
C21	0.0397 (16)	0.0394 (17)	0.0491 (16)	0.0045 (14)	0.0143 (13)	0.0086 (14)
C22	0.0489 (18)	0.0438 (18)	0.0572 (18)	0.0033 (15)	0.0155 (14)	0.0068 (15)
C23	0.0553 (19)	0.0463 (19)	0.063 (2)	0.0138 (16)	0.0242 (16)	0.0188 (16)
C24	0.0485 (18)	0.072 (2)	0.0536 (19)	0.0149 (18)	0.0167 (15)	0.0165 (18)
C25	0.0393 (16)	0.061 (2)	0.0470 (17)	0.0030 (16)	0.0125 (13)	0.0008 (15)
C26	0.0447 (16)	0.0402 (17)	0.0545 (17)	0.0030 (15)	0.0163 (14)	0.0049 (14)
C27	0.0453 (17)	0.0438 (19)	0.0538 (18)	0.0053 (16)	0.0106 (14)	0.0073 (15)
O21	0.0881 (17)	0.0404 (14)	0.0693 (14)	−0.0031 (12)	−0.0071 (12)	0.0128 (11)
O22	0.0648 (14)	0.0430 (13)	0.0590 (12)	0.0008 (11)	−0.0045 (11)	0.0028 (11)
N23	0.081 (2)	0.055 (2)	0.093 (2)	0.0222 (19)	0.0367 (19)	0.0277 (19)
O23	0.105 (2)	0.0486 (17)	0.154 (3)	0.0056 (16)	0.002 (2)	0.0118 (18)
O24	0.162 (3)	0.081 (2)	0.0900 (19)	0.050 (2)	0.0330 (19)	0.0453 (16)
N25	0.0485 (16)	0.091 (3)	0.0623 (19)	0.0074 (18)	0.0129 (14)	−0.0078 (19)
O25	0.094 (2)	0.080 (2)	0.0763 (17)	−0.0145 (17)	0.0107 (14)	−0.0151 (15)
O26	0.0714 (16)	0.134 (3)	0.0680 (16)	0.0246 (17)	−0.0131 (13)	−0.0087 (16)
C31	0.0415 (16)	0.0395 (18)	0.0516 (17)	−0.0065 (14)	0.0034 (13)	0.0010 (14)
C32	0.0388 (15)	0.053 (2)	0.0468 (16)	−0.0075 (15)	0.0045 (13)	−0.0017 (15)
C33	0.0401 (16)	0.0412 (17)	0.0525 (17)	−0.0044 (14)	0.0055 (13)	−0.0082 (14)
C34	0.0549 (19)	0.0364 (17)	0.0552 (18)	−0.0009 (15)	0.0003 (14)	−0.0012 (14)
C35	0.062 (2)	0.0405 (18)	0.0459 (17)	0.0014 (16)	0.0009 (14)	−0.0030 (14)
C36	0.0569 (19)	0.0342 (17)	0.0590 (19)	0.0008 (15)	0.0043 (15)	−0.0028 (14)
C37	0.0404 (17)	0.050 (2)	0.061 (2)	−0.0055 (16)	0.0050 (14)	0.0099 (16)
O31	0.0908 (18)	0.0440 (14)	0.0705 (14)	0.0092 (13)	0.0068 (12)	0.0120 (12)
O32	0.0626 (14)	0.0772 (17)	0.0547 (13)	0.0112 (13)	0.0042 (11)	0.0133 (12)
N33	0.0556 (17)	0.0492 (17)	0.0646 (18)	−0.0003 (14)	0.0084 (14)	−0.0133 (15)
O33	0.0948 (18)	0.0762 (17)	0.0617 (15)	0.0086 (14)	0.0136 (13)	−0.0210 (13)
O34	0.106 (2)	0.0430 (14)	0.0829 (16)	0.0133 (14)	0.0195 (14)	0.0012 (13)
N35	0.116 (3)	0.0491 (19)	0.0548 (17)	0.018 (2)	0.0041 (17)	−0.0039 (15)
O35	0.146 (15)	0.058 (5)	0.052 (5)	0.019 (7)	0.012 (7)	0.004 (4)
O36	0.116 (9)	0.047 (4)	0.069 (4)	0.013 (4)	0.009 (6)	−0.017 (3)
N45	0.116 (3)	0.0491 (19)	0.0548 (17)	0.018 (2)	0.0041 (17)	−0.0039 (15)
O45	0.122 (10)	0.067 (5)	0.056 (4)	0.025 (5)	−0.009 (5)	0.006 (4)
O46	0.171 (10)	0.064 (5)	0.062 (3)	0.038 (7)	0.026 (6)	−0.007 (3)

### Geometric parameters (Å, º)

**Table:**

C1—C2	1.380 (4)	C16—H16B	0.9700
C1—C11A	1.388 (4)	C17—H17A	0.9600
C1—H1	0.9300	C17—H17B	0.9600
C2—C3	1.364 (5)	C17—H17C	0.9600
C2—H2	0.9300	C21—C26	1.378 (4)
C3—C4	1.371 (4)	C21—C22	1.384 (4)
C3—H3	0.9300	C21—C27	1.498 (4)
C4—C4A	1.393 (4)	C22—C23	1.378 (4)
C4—H4	0.9300	C22—H22	0.9300
C4A—C11A	1.392 (4)	C23—C24	1.373 (4)
C4A—N5	1.410 (3)	C23—N23	1.469 (4)
N5—C5A	1.424 (3)	C24—C25	1.364 (4)
N5—H5	0.88 (3)	C24—H24	0.9300
C5A—C6	1.382 (4)	C25—C26	1.379 (4)
C5A—C9A	1.394 (4)	C25—N25	1.478 (4)
C6—C7	1.380 (4)	C26—H26	0.9300
C6—H6	0.9300	C27—O21	1.218 (3)
C7—C8	1.372 (4)	C27—O22	1.290 (3)
C7—H7	0.9300	O22—H22A	1.11 (4)
C8—C9	1.376 (4)	N23—O24	1.210 (4)
C8—Cl8	1.749 (3)	N23—O23	1.221 (4)
C9—C9A	1.394 (4)	N25—O26	1.220 (3)
C9—H9	0.9300	N25—O25	1.220 (4)
C9A—N10	1.404 (3)	C31—C32	1.380 (4)
N10—C11	1.287 (3)	C31—C36	1.384 (4)
C11—N11	1.394 (3)	C31—C37	1.510 (4)
C11—C11A	1.489 (4)	C32—C33	1.375 (4)
N11—C12	1.455 (3)	C32—H32	0.9300
N11—C16	1.455 (3)	C33—C34	1.378 (4)
C12—C13	1.500 (4)	C33—N33	1.472 (4)
C12—H12A	0.9700	C34—C35	1.372 (4)
C12—H12B	0.9700	C34—H34	0.9300
C13—N14	1.494 (3)	C35—C36	1.376 (4)
C13—H13A	0.9700	C35—N35	1.473 (4)
C13—H13B	0.9700	C36—H36	0.9300
N14—C17	1.490 (4)	C37—O31	1.226 (4)
N14—C15	1.495 (3)	C37—O32	1.274 (3)
N14—H14	1.00 (3)	O32—H22A	1.35 (4)
C15—C16	1.512 (4)	N33—O34	1.217 (3)
C15—H15A	0.9700	N33—O33	1.218 (3)
C15—H15B	0.9700	N35—O35	1.224 (8)
C16—H16A	0.9700	N35—O36	1.249 (6)
			
C2—C1—C11A	121.5 (3)	H15A—C15—H15B	108.0
C2—C1—H1	119.2	N11—C16—C15	111.7 (2)
C11A—C1—H1	119.2	N11—C16—H16A	109.3
C3—C2—C1	119.8 (3)	C15—C16—H16A	109.3
C3—C2—H2	120.1	N11—C16—H16B	109.3
C1—C2—H2	120.1	C15—C16—H16B	109.3
C2—C3—C4	120.1 (3)	H16A—C16—H16B	107.9
C2—C3—H3	120.0	N14—C17—H17A	109.5
C4—C3—H3	120.0	N14—C17—H17B	109.5
C3—C4—C4A	120.8 (3)	H17A—C17—H17B	109.5
C3—C4—H4	119.6	N14—C17—H17C	109.5
C4A—C4—H4	119.6	H17A—C17—H17C	109.5
C11A—C4A—C4	119.6 (3)	H17B—C17—H17C	109.5
C11A—C4A—N5	120.7 (2)	C26—C21—C22	119.2 (3)
C4—C4A—N5	119.7 (3)	C26—C21—C27	119.2 (3)
C4A—N5—C5A	117.7 (2)	C22—C21—C27	121.6 (3)
C4A—N5—H5	112.6 (18)	C23—C22—C21	118.9 (3)
C5A—N5—H5	108.5 (18)	C23—C22—H22	120.5
C6—C5A—C9A	119.3 (2)	C21—C22—H22	120.5
C6—C5A—N5	118.7 (2)	C24—C23—C22	122.7 (3)
C9A—C5A—N5	121.8 (2)	C24—C23—N23	118.8 (3)
C7—C6—C5A	121.9 (3)	C22—C23—N23	118.5 (3)
C7—C6—H6	119.1	C25—C24—C23	117.1 (3)
C5A—C6—H6	119.1	C25—C24—H24	121.4
C8—C7—C6	118.4 (3)	C23—C24—H24	121.4
C8—C7—H7	120.8	C24—C25—C26	122.1 (3)
C6—C7—H7	120.8	C24—C25—N25	119.3 (3)
C7—C8—C9	121.0 (3)	C26—C25—N25	118.5 (3)
C7—C8—Cl8	119.8 (2)	C21—C26—C25	119.9 (3)
C9—C8—Cl8	119.2 (2)	C21—C26—H26	120.1
C8—C9—C9A	120.7 (3)	C25—C26—H26	120.1
C8—C9—H9	119.7	O21—C27—O22	124.3 (3)
C9A—C9—H9	119.7	O21—C27—C21	119.5 (3)
C9—C9A—C5A	118.6 (3)	O22—C27—C21	116.2 (3)
C9—C9A—N10	115.3 (2)	C27—O22—H22A	114.6 (17)
C5A—C9A—N10	125.5 (2)	O24—N23—O23	124.1 (3)
C11—N10—C9A	124.3 (2)	O24—N23—C23	117.7 (4)
N10—C11—N11	116.5 (2)	O23—N23—C23	118.1 (3)
N10—C11—C11A	128.5 (2)	O26—N25—O25	124.8 (3)
N11—C11—C11A	114.4 (2)	O26—N25—C25	117.9 (3)
C1—C11A—C4A	118.1 (2)	O25—N25—C25	117.3 (3)
C1—C11A—C11	119.7 (3)	C32—C31—C36	119.8 (3)
C4A—C11A—C11	122.2 (2)	C32—C31—C37	120.4 (3)
C11—N11—C12	119.3 (2)	C36—C31—C37	119.7 (3)
C11—N11—C16	120.9 (2)	C33—C32—C31	119.1 (3)
C12—N11—C16	111.7 (2)	C33—C32—H32	120.4
N11—C12—C13	113.0 (2)	C31—C32—H32	120.4
N11—C12—H12A	109.0	C32—C33—C34	122.6 (3)
C13—C12—H12A	109.0	C32—C33—N33	119.4 (3)
N11—C12—H12B	109.0	C34—C33—N33	118.0 (3)
C13—C12—H12B	109.0	C35—C34—C33	116.7 (3)
H12A—C12—H12B	107.8	C35—C34—H34	121.6
N14—C13—C12	109.5 (2)	C33—C34—H34	121.6
N14—C13—H13A	109.8	C34—C35—C36	122.8 (3)
C12—C13—H13A	109.8	C34—C35—N35	118.3 (3)
N14—C13—H13B	109.8	C36—C35—N35	119.0 (3)
C12—C13—H13B	109.8	C35—C36—C31	119.0 (3)
H13A—C13—H13B	108.2	C35—C36—H36	120.5
C17—N14—C13	112.7 (2)	C31—C36—H36	120.5
C17—N14—C15	111.9 (2)	O31—C37—O32	126.1 (3)
C13—N14—C15	109.5 (2)	O31—C37—C31	118.7 (3)
C17—N14—H14	108.2 (16)	O32—C37—C31	115.2 (3)
C13—N14—H14	108.3 (16)	C37—O32—H22A	119.2 (14)
C15—N14—H14	105.9 (15)	O34—N33—O33	124.1 (3)
N14—C15—C16	111.3 (2)	O34—N33—C33	118.0 (3)
N14—C15—H15A	109.4	O33—N33—C33	117.9 (3)
C16—C15—H15A	109.4	O35—N35—O36	126.1 (11)
N14—C15—H15B	109.4	O35—N35—C35	118.6 (11)
C16—C15—H15B	109.4	O36—N35—C35	115.0 (7)
			
C11A—C1—C2—C3	−0.1 (5)	N14—C15—C16—N11	54.9 (3)
C1—C2—C3—C4	−2.2 (5)	C26—C21—C22—C23	1.2 (4)
C2—C3—C4—C4A	1.1 (5)	C27—C21—C22—C23	−179.4 (2)
C3—C4—C4A—C11A	2.1 (4)	C21—C22—C23—C24	−1.6 (4)
C3—C4—C4A—N5	−179.0 (3)	C21—C22—C23—N23	179.2 (2)
C11A—C4A—N5—C5A	−56.2 (3)	C22—C23—C24—C25	0.8 (4)
C4—C4A—N5—C5A	125.0 (3)	N23—C23—C24—C25	−179.9 (3)
C4A—N5—C5A—C6	−125.3 (3)	C23—C24—C25—C26	0.3 (4)
C4A—N5—C5A—C9A	59.5 (3)	C23—C24—C25—N25	179.0 (2)
C9A—C5A—C6—C7	3.9 (4)	C22—C21—C26—C25	−0.1 (4)
N5—C5A—C6—C7	−171.3 (2)	C27—C21—C26—C25	−179.6 (2)
C5A—C6—C7—C8	−1.0 (4)	C24—C25—C26—C21	−0.7 (4)
C6—C7—C8—C9	−2.5 (4)	N25—C25—C26—C21	−179.4 (2)
C6—C7—C8—Cl8	174.8 (2)	C26—C21—C27—O21	−7.7 (4)
C7—C8—C9—C9A	3.2 (4)	C22—C21—C27—O21	172.9 (3)
Cl8—C8—C9—C9A	−174.1 (2)	C26—C21—C27—O22	172.8 (2)
C8—C9—C9A—C5A	−0.3 (4)	C22—C21—C27—O22	−6.7 (4)
C8—C9—C9A—N10	172.0 (2)	C24—C23—N23—O24	−0.1 (4)
C6—C5A—C9A—C9	−3.2 (4)	C22—C23—N23—O24	179.2 (3)
N5—C5A—C9A—C9	171.9 (2)	C24—C23—N23—O23	177.3 (3)
C6—C5A—C9A—N10	−174.5 (2)	C22—C23—N23—O23	−3.4 (4)
N5—C5A—C9A—N10	0.6 (4)	C24—C25—N25—O26	6.9 (4)
C9—C9A—N10—C11	155.9 (2)	C26—C25—N25—O26	−174.3 (3)
C5A—C9A—N10—C11	−32.5 (4)	C24—C25—N25—O25	−173.5 (3)
C9A—N10—C11—N11	−174.9 (2)	C26—C25—N25—O25	5.3 (4)
C9A—N10—C11—C11A	−4.2 (4)	C36—C31—C32—C33	0.8 (4)
C2—C1—C11A—C4A	3.3 (4)	C37—C31—C32—C33	−175.0 (2)
C2—C1—C11A—C11	−175.9 (3)	C31—C32—C33—C34	−0.5 (4)
C4—C4A—C11A—C1	−4.2 (4)	C31—C32—C33—N33	178.2 (2)
N5—C4A—C11A—C1	176.9 (2)	C32—C33—C34—C35	−0.3 (4)
C4—C4A—C11A—C11	174.9 (2)	N33—C33—C34—C35	−179.0 (3)
N5—C4A—C11A—C11	−3.9 (4)	C33—C34—C35—C36	0.8 (5)
N10—C11—C11A—C1	−141.2 (3)	C33—C34—C35—N35	179.4 (3)
N11—C11—C11A—C1	29.6 (3)	C34—C35—C36—C31	−0.4 (5)
N10—C11—C11A—C4A	39.7 (4)	N35—C35—C36—C31	−179.1 (3)
N11—C11—C11A—C4A	−149.5 (2)	C32—C31—C36—C35	−0.4 (4)
N10—C11—N11—C12	9.7 (4)	C37—C31—C36—C35	175.5 (3)
C11A—C11—N11—C12	−162.3 (2)	C32—C31—C37—O31	177.8 (3)
N10—C11—N11—C16	−136.6 (3)	C36—C31—C37—O31	1.9 (4)
C11A—C11—N11—C16	51.4 (3)	C32—C31—C37—O32	−0.2 (4)
C11—N11—C12—C13	−94.0 (3)	C36—C31—C37—O32	−176.1 (3)
C16—N11—C12—C13	55.2 (3)	C32—C33—N33—O34	−173.7 (3)
N11—C12—C13—N14	−57.1 (3)	C34—C33—N33—O34	5.0 (4)
C12—C13—N14—C17	−177.7 (3)	C32—C33—N33—O33	5.9 (4)
C12—C13—N14—C15	57.1 (3)	C34—C33—N33—O33	−175.4 (3)
C17—N14—C15—C16	177.5 (3)	C34—C35—N35—O35	−15.5 (15)
C13—N14—C15—C16	−56.8 (3)	C36—C35—N35—O35	163.2 (15)
C11—N11—C16—C15	95.5 (3)	C34—C35—N35—O36	158.5 (11)
C12—N11—C16—C15	−53.1 (3)	C36—C35—N35—O36	−22.8 (11)

### Hydrogen-bond geometry (Å, º)

**Table:**

*D*—H···*A*	*D*—H	H···*A*	*D*···*A*	*D*—H···*A*
N5—H5···O21	0.88 (3)	2.23 (3)	3.079 (3)	162 (3)
O22—H22*A*···O32	1.11 (4)	1.35 (4)	2.453 (3)	169 (3)
C4—H4···O21	0.93	2.48	3.280 (4)	144
C13—H13*A*···O34	0.97	2.60	3.539 (4)	164
N14—H14···O31^i^	1.00 (3)	1.70 (3)	2.689 (3)	169 (3)
C1—H1···O35^ii^	0.93	2.39	3.292 (13)	164
C7—H7···O36^iii^	0.93	2.34	3.242 (13)	163
C7—H7···O46^iii^	0.93	2.37	3.253 (14)	159

Symmetry codes: (i) −*x*+3/2, *y*−1/2, −*z*+1/2; (ii) *x*−1/2, −*y*+1/2, *z*−1/2; (iii) −*x*+1, −*y*+1, −*z*+1.
